# Mathematical Modeling of Free Thyroxine Concentrations During Methimazole Treatment for Graves’ Disease: Development and Validation of a Computer-Aided Thyroid Treatment Method

**DOI:** 10.3389/fendo.2022.841888

**Published:** 2022-05-31

**Authors:** Verena Theiler-Schwetz, Thomas Benninger, Christian Trummer, Stefan Pilz, Markus Reichhartinger

**Affiliations:** ^1^Division of Endocrinology and Diabetology, Department of Internal Medicine, Medical University of Graz, Graz, Austria; ^2^Institute of Automation and Control, Graz University of Technology, Graz, Austria

**Keywords:** Graves’ disease, hyperthyroidism, mathematical thyroid model, computer-aided treatment, biomedical modeling

## Abstract

**Background:**

Methimazole (MMI) is the first-line treatment for patients with Graves’ disease (GD). While there are empirical recommendations for initial MMI doses, there is no clear guidance for subsequent MMI dose titrations. We aimed to (a) develop a mathematical model capturing the dynamics of free thyroxine (FT4) during MMI treatment (b), validate this model by use of numerical simulation in comparison with real-life patient data (c), develop the software application Digital Thyroid (DigiThy) serving either as a practice tool for treating virtual patients or as a decision support system with dosing recommendations for MMI, and (d) validate this software framework by comparing the efficacy of its MMI dosing recommendations with that from clinical endocrinologists.

**Methods:**

Based on concepts of automatic control and by use of optimization techniques, we developed two first order ordinary differential equations for modeling FT4 dynamics during MMI treatment. Clinical data from patients with GD derived from the outpatient clinic of Endocrinology at the Medical University of Graz, Austria, were used to develop and validate this model. It was subsequently used to create the web-based software application DigiThy as a simulation environment for treating virtual patients and an autonomous computer-aided thyroid treatment (CATT) method providing MMI dosing recommendations.

**Results:**

Based on MMI doses, concentrations of FT4, thyroid-stimulating hormone (TSH), and TSH-receptor antibodies (TRAb), a mathematical model with 8 patient-specific constants was developed. Predicted FT4 concentrations were not significantly different compared to the available consecutively measured FT4 concentrations in 9 patients with GD (52 data pairs, p=0.607). Treatment success of MMI dosing recommendations in 41 virtually generated patients defined by achieved target FT4 concentrations preferably with low required MMI doses was similar between CATT and usual care. Statistically, CATT was significantly superior (p<0.001).

**Conclusions:**

Our mathematical model produced valid FT4 predictions during MMI treatment in GD and provided the basis for the DigiThy application already serving as a training tool for treating virtual patients. Clinical trial data are required to evaluate whether DigiThy can be approved as a decision support system with automatically generated MMI dosing recommendations.

## 1 Introduction

Graves’ disease (GD) is the most common cause of hyperthyroidism with a lifetime risk of 3% for women and of 0.5% for men (1). Patients with GD can be treated with antithyroid drugs (ATDs), radioactive iodine (RAI) ablation, and thyroidectomy, with the preferred initial treatment modality being the ATD methimazole (MMI) (2–16). Even in countries with a traditionally high use of RAI clinical practice is shifting towards ATD treatment (17, 18).

ATD treatment can follow either the titration regimen with ATD dose titration according to thyroid function tests to achieve euthyroidism, or the block and replace regimen. The majority of GD patients are treated with the titration regimen (5, 9). Initial MMI doses are typically based on the severity of hyperthyroidism. A rough guide to initial daily dosing is: 5-10 mg MMI if free thyroxine (FT4) is 1-1.5 times the upper limit of normal, 10-20 mg MMI if FT4 is 1.5-2 times the upper limit of normal and 30-40 mg MMI if FT4 is 2-3 times the upper limit of normal (2). FT4 and free triiodothyronine (FT3) should be measured 2 to 6 weeks after starting MMI treatment and some experts suggest to reduce the MMI dose by 30% to 50% once euthyroidism is achieved (2, 3). Apart from this, there is no clear guidance for further MMI dose titration to achieve euthyroidism except using the smallest possible dose of ATDs and withdrawing ATDs after 12 to 18 months of treatment in case of euthyroidism and normal thyroid-stimulating hormone (TSH) receptor antibodies (TRAb) (2, 3).

In light of millions of patients on ATD therapy worldwide, the relatively arbitrary dose titration requiring regular visits with thyroid experts, as well as high relapse rates (19), there is a great need for systematic and computer-aided MMI treatment procedures to ensure fast and reliable desired thyroid hormone concentrations in patients suffering from GD. As such control methods often rely on mathematical models (e.g. for the purpose of simulation and of controller synthesis), a well-justified mathematical model is a key component for the design of a systematic and possibly automated treatment framework. When developing these mathematical models, a variety of factors relevant for thyroid hormone regulation, among them MMI effects and enzyme kinetics, must be taken into account (16, 20–25). From a clinical point of view, a mathematical model in combination with a treatment algorithm may be used for the construction of software devices (e.g. web and cell phone applications used by both patients and endocrinologists) which serve as a highly personalized digital thyroid treatment system able to improve patient care.

Previous attempts to model FT4 concentrations during ATD treatment are sparse [see e.g (24, 25)]. and do not provide a useful basis for a reliable design of model-based computer-aided thyroid treatment (CATT) systems in GD. As for hypothyroidism, one online available simulation software for thyroid hormone replacement therapy is the THYROSIM app, but such an app is not yet available for ATD therapy (26). Therefore, the aim of this study is, firstly, to establish a mathematical model based on the Michaelis-Menten-enzyme kinetics [see e.g (27)]. that is able to reproduce the time evolution of FT4 during MMI treatment, i.e. to predict future fT4 concentrations. Secondly, we aim to validate this model by use of numerical simulation in comparison to real-life data with GD patients derived from the outpatient clinic of Endocrinology at the Medical University of Graz, Austria. Thirdly, this model is used to create the web-based software application Digital Thyroid (DigiThy) in order to provide both a simulation environment for treating virtual patients and a CATT system that may serve as a future decision support system with dosing recommendations for the treatment of GD. Fourthly, we aim to validate the CATT method by comparing its dosing recommendations with those from clinical endocrinologists. For this purpose, we use patients with GD who were virtually generated by DigiThy. The comparison between treatments is based on treatment success (efficacy) defined as achievement of target FT4 concentrations and minimization of MMI doses.

## 2 Materials and Methods

### 2.1 Development of the Mathematical Model

The mathematical model presented is based on concepts of automatic control (28, 29). It consists of two first order ordinary differential equations including eight patient-specific constant parameters and three options for its excitation, i.e., the stimulation by MMI, TRAb and TSH. In order to identify reasonable intervals for each of the eight patient-specific parameters, methods of constraint optimization in combination with real patient data and numerical simulations of the model were applied. More specifically, simulations with a different choice of patient-specific parameters selected out of predefined intervals were carried out. The parameters achieving the most accurate match between the simulated and the real patient FT4 evolution were used to characterize the considered individual patient. This procedure was realized for all available patient data using the nonlinear least squares implementation in the Optimization Toolbox™ of the MATLAB^®^ software. The presented model in combination with its eight parameters is able to generate fictitious FT4 time evolutions of virtual patients. This is realized by selecting specific values for each of the eight parameters and subsequently using them in a numerical simulation study, where the MMI dosage can either be prescribed manually (i.e., “usual care”) or is computer determined (i.e., “computer-aided”). For this purpose, the model and the developed automated CATT algorithm were implemented in MATLAB^®^/Simulink^®^, and for its realization within DigiThy in the Octave^®^ software.

### 2.2 Real Patient Data

Real patient data were derived from study participants of the Graz Endocrinology Registry Study that includes patients who are routinely treated in the outpatient clinic of the Division of Endocrinology and Diabetology, Department of Internal Medicine, Medical University of Graz, Austria. The study was approved by the ethics committee at the Medical University of Graz, Austria, and all study participants gave written informed consent before being involved in any study-related procedures. All available Registry patients diagnosed with GD and routinely treated with MMI were included in our study. Additional criteria for selection were available TRAb concentrations at baseline with at least one follow-up measurement, and consistent availability of fT4 and TSH data as well as prescribed MMI dosages throughout the follow-up appointments at the outpatient clinic.

### 2.3 Laboratory Measurements

Serum FT4 (reference range: 13–23 pmol/l), FT3 (reference range: 3.1–6.8 pmol/l), and TSH (reference range: 0.27–4.2 µU/mL) were determined by direct chemiluminescence technology on an ADVIA Centaur XP automated analyser (Siemens Healthcare Diagnostics Inc., Tarrytown, NY, U.S.A.). TRAb (reference range: 0–15.0 U/L) were determined by an enzyme-linked immunosorbent assay (ELISA) (IASONTRAB^®^, IASON GmbH, Graz, Austria).

### 2.4 Statistical Analyses

Continuous data with a normal distribution are shown as means with standard deviation, variables with a skewed distribution are shown as medians with interquartile ranges. Group comparisons were performed by a paired Student’s t-test for normally distributed data and by a Wilcoxon test in case of a non-normal distribution. A p-value <0.05 was considered statistically significant. All statistical analyses were performed using SPSS version 27.0 software (SPSS, Chicago, IL).

## 3 Results

According to our inclusion criteria based on data availability, we retrieved 41 patients from the Graz Endocrinology Registry Study, who were thus included into the present investigation. We developed a new dynamic model capturing the main dynamics of the FT4-production of a thyroid stimulated by TSH, TRAb and MMI-dosing (see Section 3.1). Time evolutions of FT4 of GD patients obtained by numerical simulation of the derived model were compared to real patient data (see Section 3.2). The developed model was used to realize a CATT framework (see Section 3.3) serving as a decision support system with dosing recommendations for MMI treatment and enabling automatic treatment of patients. Finally, in Section 3.4, we validated this software framework in virtually generated patients by comparing its treatment success defined as achievement of target FT4 concentrations preferably with low MMI doses with that from clinical endocrinologists.

### 3.1 Mathematical Model

The dynamics of the amount of MMI within the thyroid gland and the FT4 concentration are assumed to be governed by the differential equations


$$\frac{dx_{1}}{dt}=k_{d}(u-x_{1})$$

$$\frac{dx_{2}}{dt}=\frac{k_{a,1}d_{1}}{d_{1}+k_{a,2}\left(1+\frac{x_{1}}{k_{m}}\right)}+\frac{k_{T,1}d_{2}}{d_{2}\left(1+\frac{d_{1}}{k_{T,2}}\right)+k_{T,3}(1+\frac{x_{1}}{k_{m}})}-k_{f}\;x_{2}$$

where the state variables *x*_1_ and *x*_2_ denote the total mass of MMI within the thyroid gland and the FT4-concentration, respectively. The input *d*_1_ of the mathematical model represents the TRAb and the input *d*_2_ denotes TSH, which are the two stimulating substances. Both TSH and TRAb stimulate the production of FT4, a process which is modeled by well-known Michaelis-Menten kinetics (27). Of note, even when TSH is starting to rise in the course of GD, it has been shown by comparison of simulation results with real patient data that the presence of TRAb has a blocking impact on the stimulation by TSH in our model. This is a reasonable modeling assumption, since both TSH and TRAb activate the TSH-receptor (30). This impact is taken into account by the occurrence of *d*_1_ within the FT4-excitation due to *d*_2_ in the second equation. The input *u* is the daily MMI dose, which, from a control engineering point of view, represents the manipulating variable. In the case of zero input, i.e., *u* = *d*_1_ = *d*_2_ = 0, the degradation of the state variables is assumed to be a linear process with the positive parameters *k_d_* and *k_f_*. The FT4 stimulus is divided into two Michaelis-Menten terms. The influence of MMI on *x*_2_ (the FT4 concentration) *via* the state variable *x*_1_ (the total mass of MMI within the thyroid gland) is scaled by the positive parameter *k_m_* and weighted by the positive constants *k_a_*_,2_ and *k_T_*_,3_.

All time-dependent variables are substances and hence


$$x_{1}(t)\geq 0,x_{2}(t)\geq 0,d_{1}(t)\geq 0,d_{2}(t)\geq 0\text{ and }u(t)\geq 0\forall t\geq 0.$$

Note that the structure of the mathematical model remains the same for any modeled GD patient. However, each patient is assumed to be characterized by its eight individual positive and constant parameters (see **Table 1**). These parameters and its corresponding intervals (lower and upper bounds) have been determined using the optimization based parameter identification approach as described above. Importantly, the eight parameters of the mathematical model are determined such that a numerical simulation generates an FT4 behavior approximating the available measured patient data, so that future FT4 concentrations can be predicted by the model when data on TSH, FT4, TRAb and MMI doses are available. It is important to note that the admissible range of parameters to be chosen has been set such that FT4 evolutions converging towards unrealistic high values cannot occur in principle. This is ensured by the properly formulated optimization problem’s constraints.

**Table 1:** Identified parameter intervals for the parameters required within the developed mathematical model.

Parameter	Unit	Lower bound	Upper bound	Short description
*k_a_*_,1_	$\frac{pM}{dayL}$	2.8881	27.7259	Maximum synthesis speed due to TRAb
*k_a_*_,2_	$\frac{U}{L}$	1.0	500	Individual TRAb impact
*k_T_*_,1_	$\frac{pM}{dayL}$	2.8881	8.6643	Maximum synthesis speed due to TSH
*k_T_*_,2_	$\frac{U}{L}$	1.0	500	TRAb blocking impact on TSH-synthesis
*k_T_*_,3_	$\frac{\mu U}{mL}$	0.1	50	Individual TSH impact
*k_f_*	$\frac{1}{day}$	0.077	0.1733	FT4 degradation rate
*k_d_*	$\frac{1}{day}$	0.1386	1.3863	Individual MMI impact
*k_m_*	*mg*	1.0	200	Individual MMI impact on FT4 production

### 3.2 Model Assessment *via* Comparison to Real Patient Data

**Figure 1:**
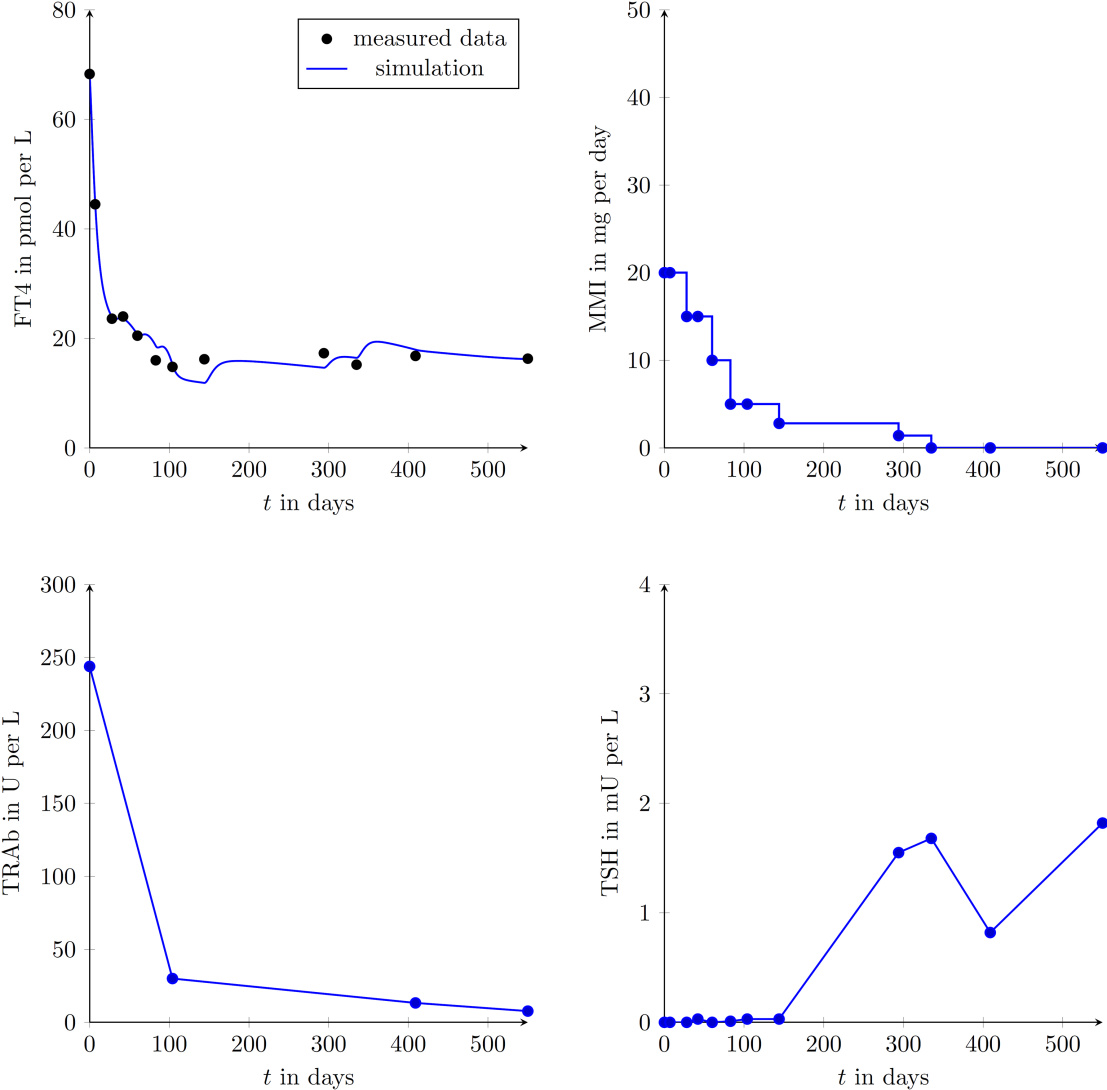
Graphical comparison of a single GD patient (female, aged 42 years at presentation) using the measured data points and the simulated trajectories obtained with the developed mathematical model. FT4, TRAb, TSH and the MMI-dosage are shown in the corresponding plots. Follow-up appointments are marked by plotted circles and it is assumed that between appointments MMI doses were taken as prescribed. During the simulation, the intervening data for TRAb and TSH were linearly interpolated.

**Figure 2:**
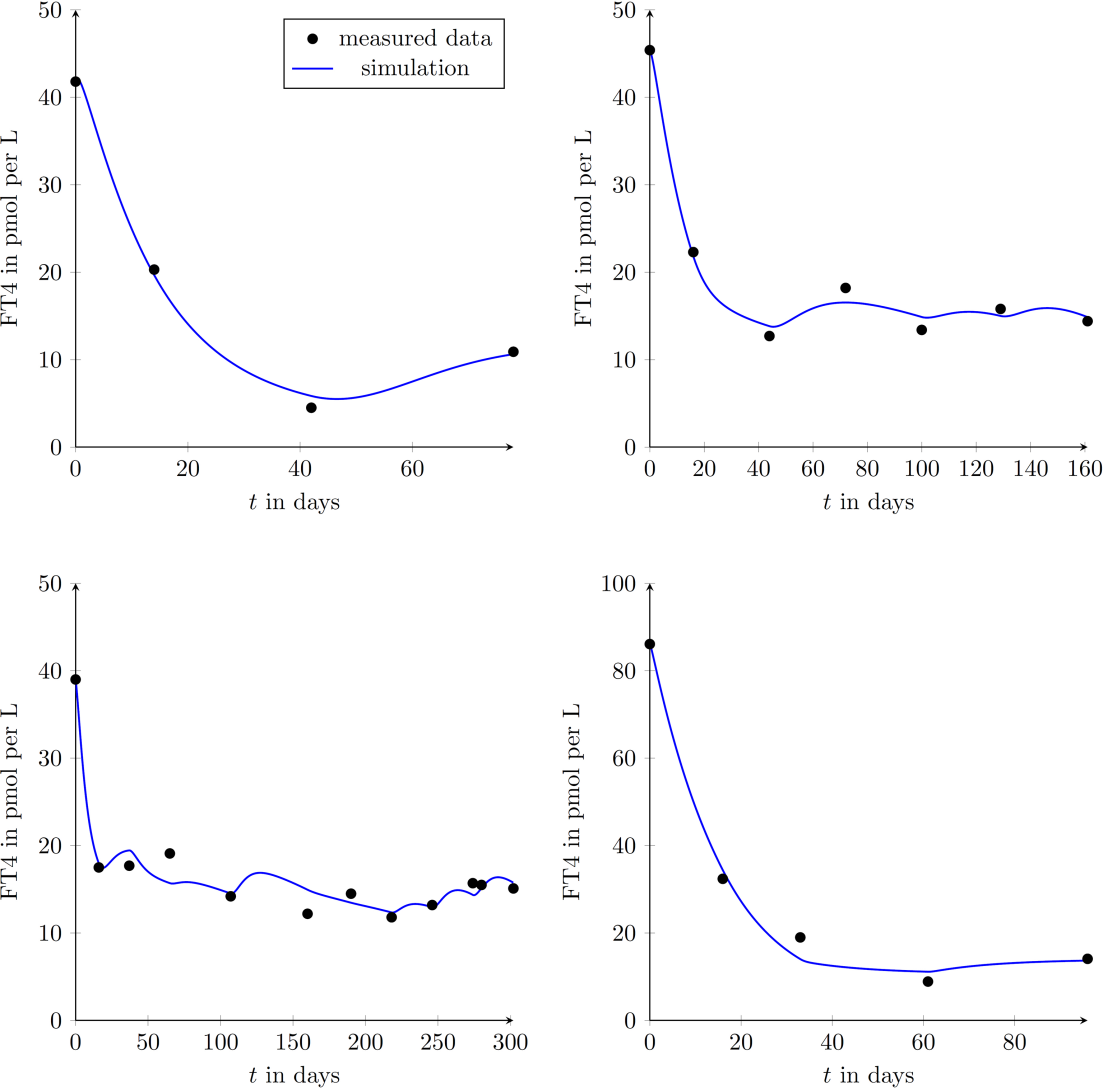
Graphical comparison between measured data points of GD patients and results obtained by simulating the mathematical model for four different treatment-naive patients (upper left diagram: female, aged 50 years; upper right diagram: female, aged 27 years; lower left diagram: female, aged 49 years; lower right diagram: female, aged 44 years;). This demonstrates the ability of reproducing different GD-courses exploiting the proposed model.

**Figure 3:**
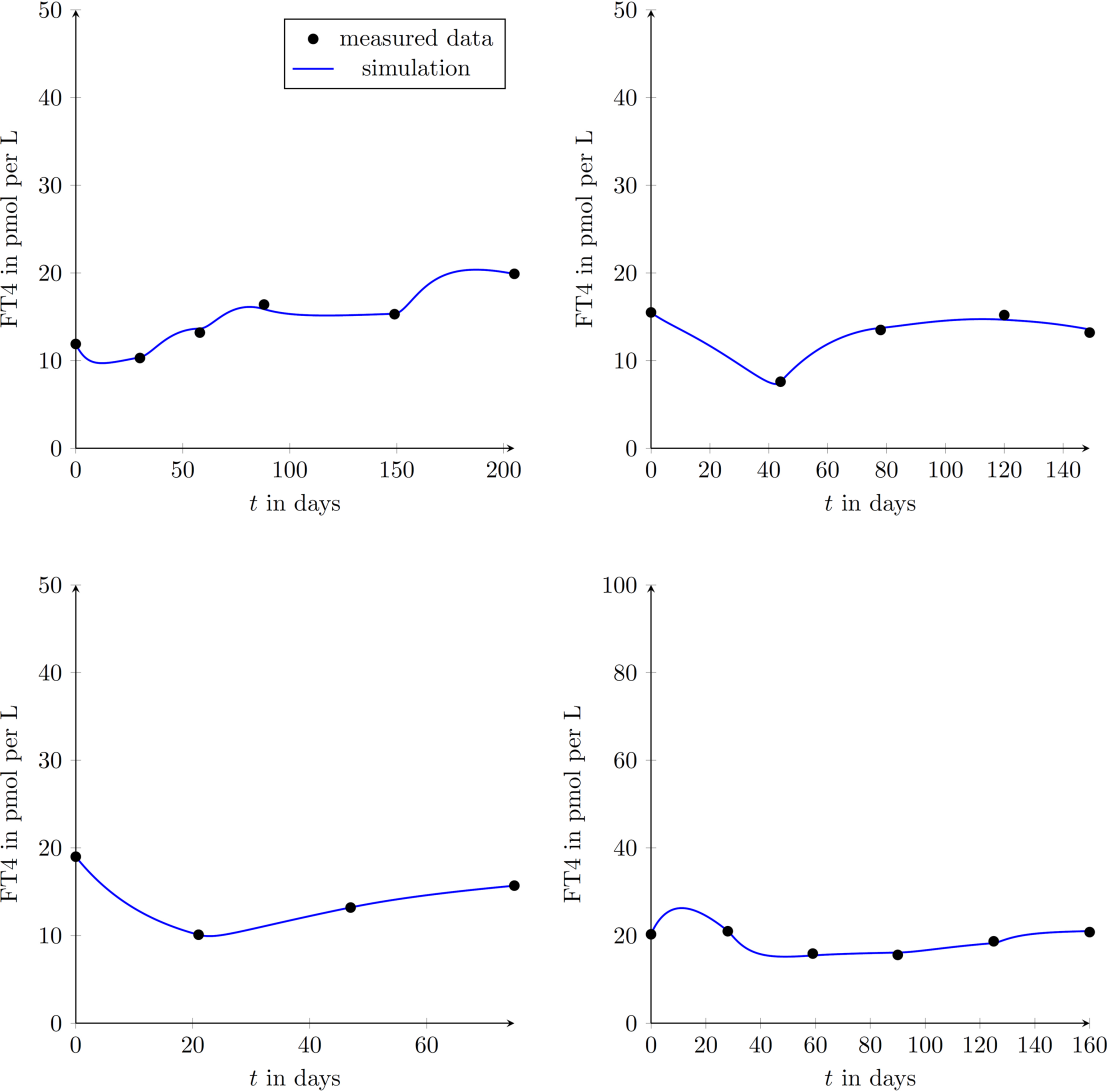
Graphical comparison between measured data points and simulated trajectories according to the mathematical model in four different patients (upper left diagram: female, aged 33 years; upper right diagram: male, aged 59 years; lower left diagram: female, aged 36 years; lower right diagram: female, aged 68 years). These patients had already been treated with MMI at inclusion into the study.

We enrolled 9 GD patients (1 man, 8 women, mean age of 45.3 ± 12.9 years) into our study (see **Table 2**). To illustrate that the presented model is in fact effectively able to capture the main dynamics of GD during MMI-treatment, TRAb stimulation and even TSH excitation, the time evolution of FT4 of a GD patient obtained by simulation is compared to real patient data (see **Figure 1**). This result obviously requires to properly select the eight patient specific constant model parameters. As mentioned above, this is realized by solving a least squares optimization problem. There, at time instances where measured FT4 data is available the squared deviation to its corresponding simulated FT4 data is determined. The eight parameters are automatically adjusted by the optimization procedure such that this deviation is minimized. Further, the input variables TRAb, TSH and the MMI-dosage are shown in the corresponding plots. **Figure 2** compares FT4 simulation results of four additional patients, who were all treatment-naive at baseline. From these illustrative comparisons it is evident that the model is able to adequately represent the dynamics of the disease. **Figure 3** shows the courses of four patients with GD already on MMI treatment upon inclusion into the Graz Endocrinology Registry Study. As illustrated in this figure, the model is also able to reproduce FT4 dynamics without knowledge of the initial MMI treatment.

**Table 2:** Selected baseline characteristics of all 9 study participants.

Parameter	Mean/median/numbers
Age (years)	45.3 ± 12.9
Gender	8 female, 1 male
Smokers	2 smokers, 2 with unknown smoking status, 5 non-smokers
Thyoid volume (mL)	Females: 17.6 ± 8.1Male: 18.1
TSH (mcU/mL)	0.0 (0.0-0.0)
fT4 (pmol/l)	44.5 ± 26.0
fT3 (pmol/L)	17.4 ± 10.8
TRAb (U/L)	108.8 ± 120.8

Data are presented as means with standard deviation, medians with interquartile ranges or numbers. Thyroid volume was available in 8 patients. TSH, thyroid-stimulation hormone; FT4, free thyroxine; FT3, free triiodothyronine; TRAb, thyrotropin receptor antibodies.

**Figure 4:**
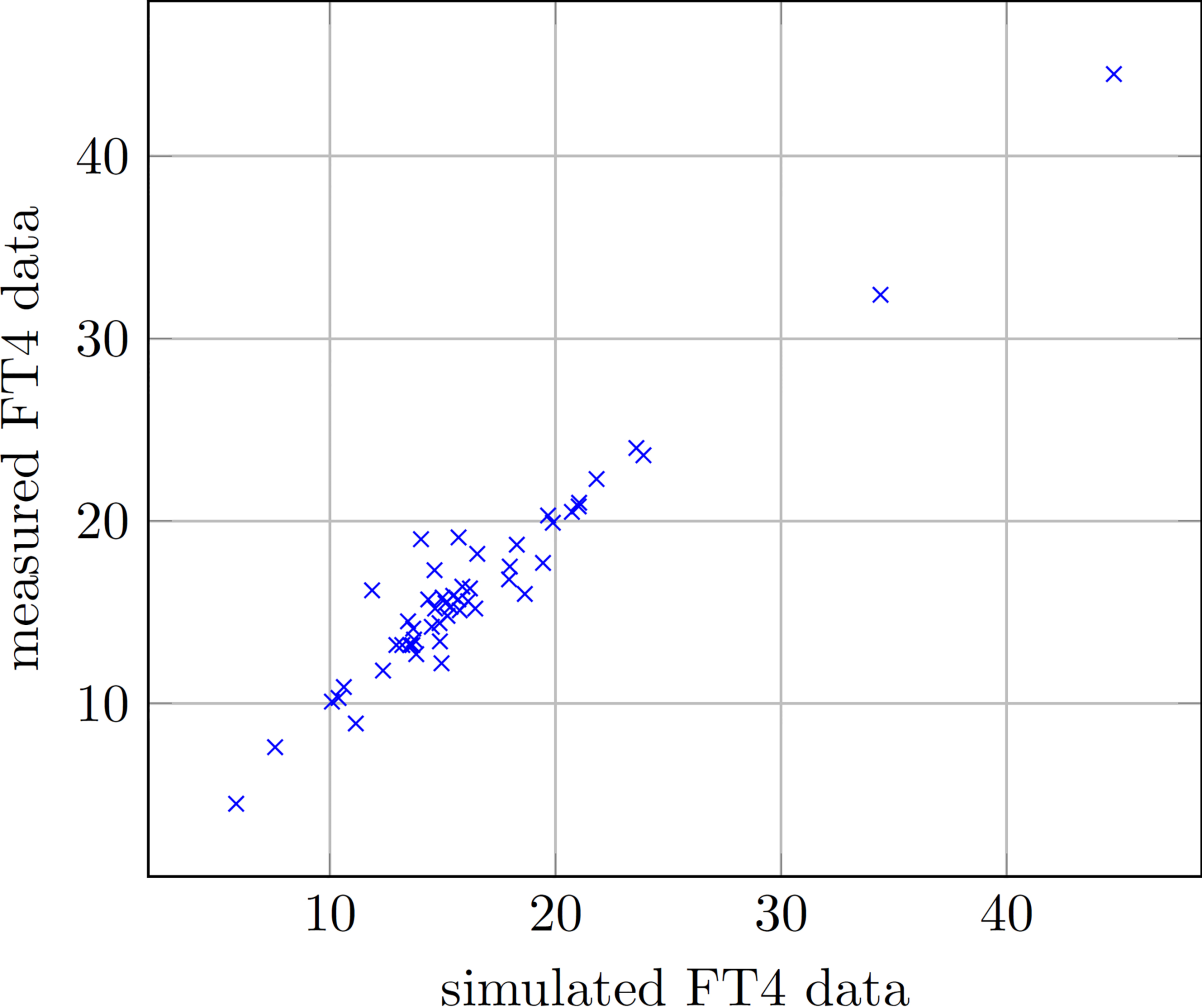
Scatter plot displaying measured and simulated FT4 data for all 9 patients taken together.

The comparison between measured and simulated (i.e. predicted) FT4 values (52 available data pairs) in all 9 patients shows no statistically significant difference (Wilcoxon test, p=0.607). The correlation between measured and simulated FT4 values was highly significant (Spearman’s rho=0.882, p<0.001, see **Figure 4**).

### 3.3 Computer Aided Thyroid Treatment Framework

The established mathematical model was implemented in software packages to perform numerical simulation studies. After the selection of the above mentioned parameters out of the predefined intervals (see **Table 1**) and the appropriate functions for the model inputs TSH and TRAb, an artificial patient treatment could be simulated. A convenient and easy-to-use web-based software application is provided at thyroid.tugraz.at and referred to as Digital Thyroid (DigiThy). Dependent on the level of automation, DigiThy can thus serve as a computer-based learning platform for physicians inexperienced in GD treatment, assist physicians by providing MMI-dose recommendations, or it could function as a fully automated CATT. The results presented in this paper are based on a fully automated CATT algorithm, i.e., it is assumed that the MMI dose computed by the CATT algorithm is taken by the patient without any modification by a physician.

#### 3.3.1 Computer Aided Thyroid Treatment Algorithm

A tailored discrete-time proportional-integral controller-based dosing strategy is implemented as a CATT-algorithm (1, 2). The controller’s task is to select the daily drug dose *u* in mg per day so that the measured FT4 dose converges into the reference interval. By design, the algorithm considers the maximum possible MMI dosage of 40 mg per day as well as the naturally occurring limit from below, i.e., 0 mg per day. Hence, any daily dosage determined by the CATT-algorithm satisfies 0 ≤ *u* ≤ 40. Since MMI is taken in the form of tablets, *u* also cannot take arbitrary values within this interval. The smallest practically relevant dose is a quarter of a 20 mg tablet, which is equivalent to 5mg MMI, which might be prescribed every other day or even every 4 days. The algorithm’s determined dosing is thus an integer multiple of 1.25mg, ensuring clinically relevant doses only. Furthermore, analyses of real treatment courses showed that abrupt changes in dosage might lead to large fluctuations in FT4-levels, possibly evoking values outside the reference intervals. To counteract these effects and ensure a gentle treatment, the algorithm limits the rate at which the dosage prescription is changed between two control appointments. The maximum rate of change depends on the previous treatment episode, i.e., the measured FT4-values. Time spans between follow-up appointments as defined by the treating physician depend on severity and development of hyperthyroidism and are obviously not constant. From a control engineering point of view, the CATT-algorithm is therefore designed to handle varying durations between follow-up appointments.

#### 3.3.2 Results Obtained by Computer Aided Thyroid Treatment

In order to test the designed CATT-algorithm in simulation studies, so-called virtual patients are generated randomly. This is realized by a feasible selection of the model parameters such that in the case of reasonable MMI-dosages, pre-specified FT4 and TRAb intervals cannot be exceeded. Furthermore, an artificial TRAb time evolution is generated for stimulating the virtual thyroid gland. The type of TRAb generation is randomly selected based on its occurrence in real GD patients (19), that of a persistent TRAb excitation being 10%, of a disappearing excitation 76%. For the remaining excitation occurrence so-called complex changes are generated.

In **Figure 4** illustrative CATT of two different virtual patients are shown. Both studies were configured such that after a duration of 28 days a new MMI dosage was allowed to be prescribed, i.e., the daily dosage remained constant for 28 days.

**Figure 5:**
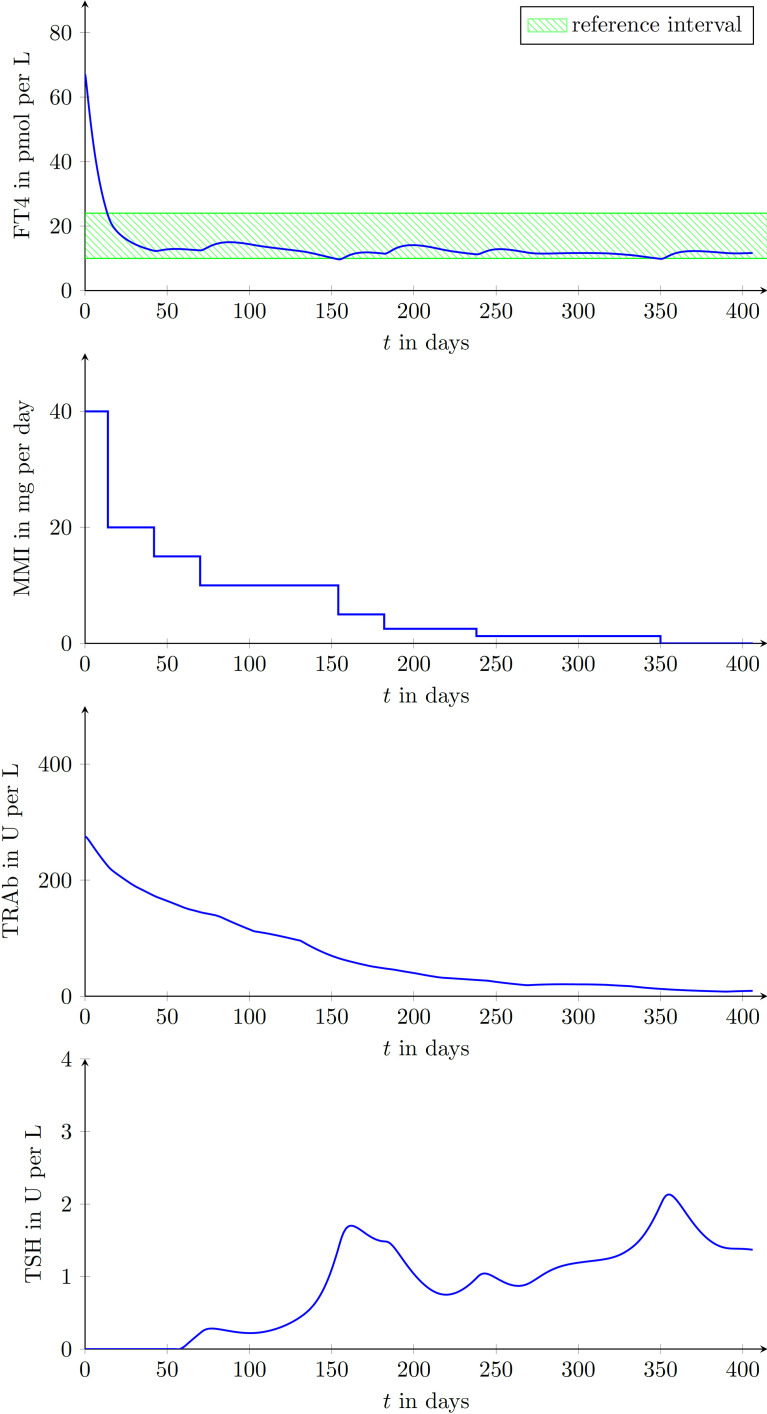
Laboratory parameters of a virtual patient treated by the proposed CATT-algorithm implemented within the DigiThy software framework. The patient shows a vanishing TRAb behavior. The proposed algorithm determines dosages such that FT4 converges into the reference interval.

The result of the first treatment (see **Figure 5**) shows a gradual reduction of the prescribed MMI-dose to keep FT4 within the reference range (in green). The evolution of TRAb declines in the course of the treatment. At the end of the treatment, i.e., after 350 days, no relapse occurs and TSH resumes its desired reference range.

**Figure 6:**
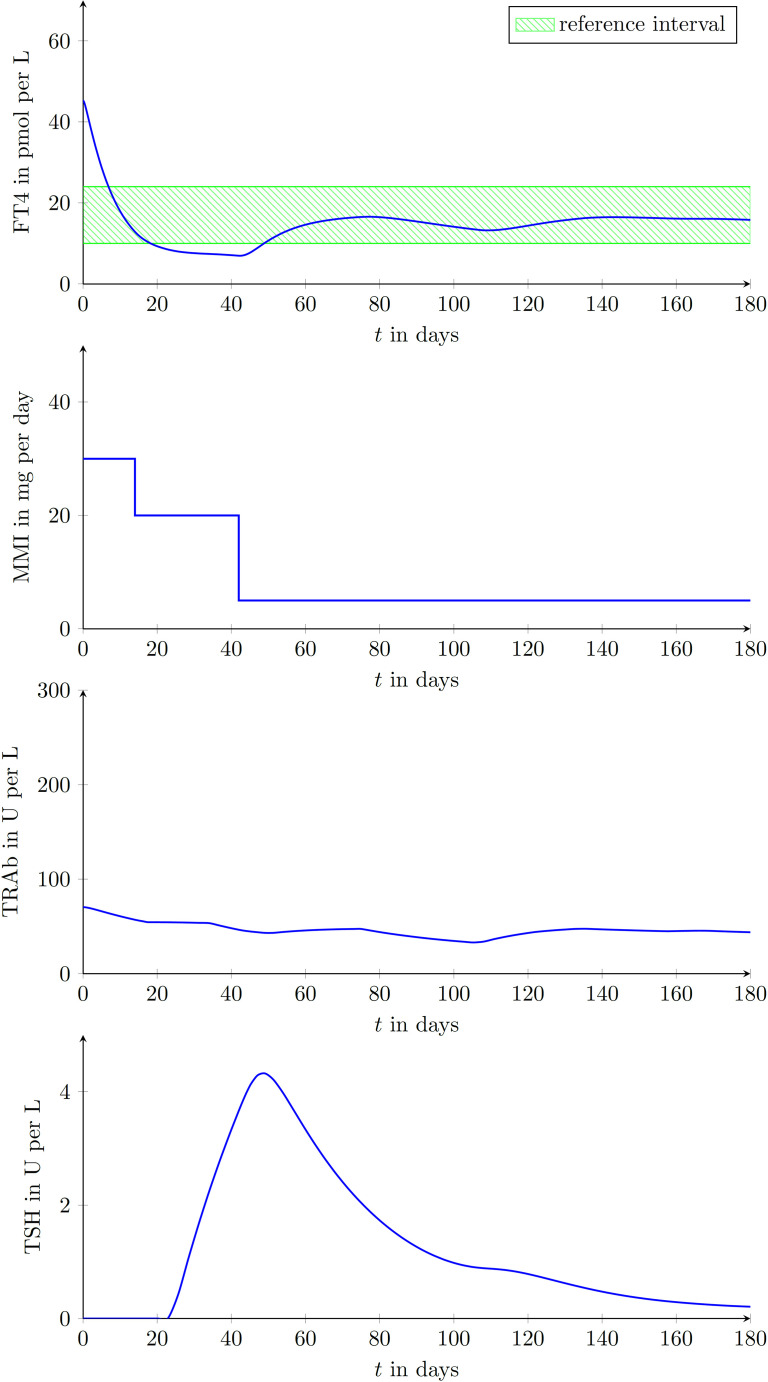
Laboratory parameters of another virtual patient treated by the proposed CATT-algorithm implemented within the DigiThy software framework. This patient shows an almost constant TRAb excitation. The proposed algorithm determines dosages such that FT4 converges into the reference interval.

In the simulated course of the treatment shown in **Figure 6**, TRAb remain persistently high and higher MMI doses were required to reach the desired FT4 reference interval.

### 3.4 Comparison of MMI Treatment by DigiThy Versus Clinical Endocrinologists

To compare MMI doses prescribed by experienced endocrinologists (i.e. usual care according to best clinical practice knowledge) with that proposed by CATT, 41 virtual patients and their respective “digital clones” were generated. In detail, by using the DigiThy application, the three clinical endocrinologists of this publication made MMI dosing recommendations based on available data for previous MMI dose, TSH, fT4 and TRAb concentrations. This process was reiterated for each patient appointment, which was mostly scheduled on a monthly basis continuing for an overall treatment period of 439 ± 206 days. CATT used the same follow-up intervals as specified by the endocrinologist for better comparability. The end of the treatment was the same for both approaches.

To assess and compare the two treatment approaches, the cost function


$$J(u,x_{2})=\sqrt{\frac{1}{N}\sum_{i=1}^{N}\left\{\alpha_{1}{\left[x_{2}(T_{i})-r\right]}^{2}+\alpha_{2}u{\left(T_{i}\right)}^{2}+\beta_{1}h_{1}\left(x_{2}(T_{i})\right)+\beta_{2}h_{2}\left(x_{2}(T_{i})\right)\right\}}$$

with the function


$$h_{1}(x_{2})={\left[max(m,x_{2})-x_{2}\right]}^{2}\;\text{and}\;h_{2}(x_{2})={\left[min(M,x_{2})-x_{2}\right]}^{2}$$

and the positive number *N* representing the number of appointments was used. In general, a cost function produces a positive real number where a low value is associated with a good performance of a treatment and vice versa. In the above presented cost function the time *T_i_* with *i* = 1, … , *N* are the time instances where an appointment took place. The positive constant weights *α*_1_, *α*_2_, *β*_1_ and *β*_2_ are positive scaling parameters whose impact will be described below. Note that the functions *h*_1_ and *h*_2_ are introduced in order to penalize hypothyroidism and hyperthyroidism separately by adjusting the positive constants *m* and *M*, respectively. The overall treatment aim is to select the dose *u* in a way that each patient achieves an FT4 concentration within a predefined reference interval with an upper bound *B_u_* and a lower bound *B_l_*. It is reasonable to define the desired concentration as $r=\frac{B_{u}+B_{l}}{2}$ . The presented results are based on *B_u_* = 24 pmol/L and *B_l_* = 9.5 pmol/L, hence *r* = 16.75 pmol/L. The first term within the cost function *J* penalizes the difference between the FT4 concentration and the desired reference concentration *r*. The factor *α*_1_ describes how strong the penalty for this difference is taken into account in the remaining terms in the cost function. Hence, the higher this value is (in comparison to the remaining weighting parameters), the more weight is given to this deviation, which is considered more important for obtaining a successful treatment. The second term in the cost function penalizes the prescribed MMI dose. The lower the prescribed dose, the smaller the penalty is. The parameter *α*_2_ describes the importance of the amount of prescribed MMI in contrast to the achieved FT4 behavior.

The achieved performances of usual care treatment and CATT were evaluated using the defined cost function. In order to consider different assessment aspects, three settings of the cost function weights *α*_1_, *α*_2_, *β*_1_ and *β*_2_ were investigated.

- Comparison based on tracking performance only (indicated by *J_u_*_,1_ for usual care, by *J_d_*_,1_ for CATT): This comparison only focuses on the quality of the treatment in terms of achieving a certain constant desired FT4 value, i.e., no further quantities are considered. The chosen parameters are *α*_1_ = 1 and *α*_2_ = *β*_1_ = *β*_2_ = 0. Therefore, deviations from the reference r=16.65 are penalized only.
- Comparison based on dosing and tracking penalties (indicated by *J_u_*_,2_ for usual care, by *J_d_*_,2_ for CATT): The chosen parameters are *α*_1_ = 1, *α*_2_ = 0.05 and *β*_1_ = *β*_2_ = 0. Therefore, any deviation from the reference *r* is penalized and, additionally, less MMI-dosage leads to less penalty. Hence, a treatment with less MMI consumption is likely to perform better in comparison to a treatment using a high dosing approach.
- Comparison based on FT4 target range (indicated by *J_u_*_,3_ for usual care, by *J_d_*_,3_ for CATT): The chosen parameters are *α*_1_ = *α*_2_ = 0 and *β*_1_ = *β*_2_ = 1 and hypothyroidism and hyperthyroidism are considered by *m* = 14.5 and *M* = 19. Therefore, only FT4 concentrations higher than 19 and lower than 14.5 $\frac{pmol}{L}$ are penalized.

**Figure 7:**
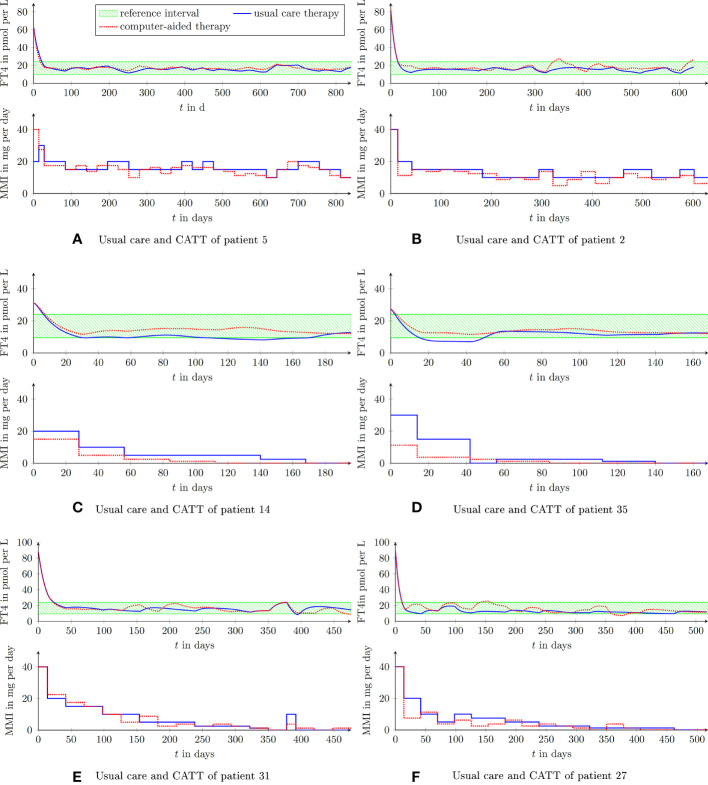
Comparison of virtual patients treated with usual care (carried out by an endocrinologist) and the proposed CATT procedure. **(A–F)** show the time evolution of FT4 and MMI dosing of selected virtual patients.

Overall, the achieved performances of CATT in terms of all three cost functions investigated were significantly lower, i.e. more favorable, than those of usual care (see **Supplementary Table 1**). In detail, *J_u_*_,1_ was 5.127 (3.460-5.511), *J_d_*_,1_ was 3.894 (2.716-4.615), p<0.001. *J_u_*_,2_was 5.705 ± 1.217, *J_d_*_,2_ was 4.609 ± 1.297, p<0.001; *J_u_*_,3_ was 2.943 ± 1.300, *J_d_*_,3_ was 2.096 ± 1.210, p<0.001. Of note, even the worst performance of the proposed CATT suggests very similar doses of MMI in comparison to usual care. In addition, no unrealistic behavior with reference to dosing recommendations and FT4 concentrations was produced by DigiThy in these 41 patients as evaluated by review of the clinical endocrinologists of this publication. In the cases of patients 5, 14 and 35 the CATT yields lower costs as compared to usual care. Note that only two out of 41 automated treatments (examples of 6 patients are shown in **Figures 7A–F**) were evaluated inferior compared to usual care, namely patient 2 (see **Figure 7B**) and patient 31 (see **Figure 7E**). Five patients (patient 2, 3, 27, 31 and 40) were treated inferior compared to usual care when using the setting of the cost function based on FT4 target range, hence the dosage is not penalized and only FT4 concentrations above 19 and under 14.5 pmol/L produce costs. The time evolution of three of these treatments are plotted in the **Figures 7B, E, F**.

## 4 Discussion

We developed a mathematical model that yielded valid predictions of FT4 concentrations in patients with GD, before and during the course of MMI treatment. This model provided the basis for the development of the web-based DigiThy training tool but may also function as a decision support system or even as a CATT method if further validated in clinical trials. In virtually generated GD patients, MMI dosing recommendations as suggested by our mathematical model were even more accurate in terms of certain performance criteria compared to those of experienced endocrinologists. Further, the model seems to be safe from a clinical point of view, as no obvious unrealistic dosing recommendations or FT4 concentrations were produced by DigiThy when treating virtual patients.

This pilot study on the development of a mathematical model and a web application (DigiThy) to guide MMI treatment in GD addresses a largely unexplored topic at the interface between mathematical modeling and clinical patient care. As MMI dosing recommendations lack a clear and easy-to-follow guideline for clinicians, current practice might be considered arbitrary and is often dependent on expert knowledge, demonstrating the clinical need to improve this situation.

That our model is capable of doing this is depicted by its ability to reproduce the FT4 evolution during an entire course of GD for a variety of different GD scenarios. More precisely, it can e.g. handle the most common types of TRAb stimulations and includes eight constant parameters to take into account patient-dependent characteristics. Further, future predictions of FT4 concentrations were extremely well comparable to the actual laboratory measurements at that time point, supported by the finding that no significant difference between measured and predicted FT4 concentrations could be found.

In contrast to our model, the few existing published dynamic models also designed to approximate thyroid hormones over time in GD are either not able to reproduce the entire, and for a treatment relevant, duration of the disease (24), provide MMI dosing simulation for non-practically relevant dosing (25) or are too detailed and complex to serve as a basis for the design of a CATT (31). Note that other existing dynamic thyroid models are largely not designed to make MMI dosing recommendations (32). Although we initially developed our model based on the input of the concentrations of FT4, TRAb and TSH, our model is also capable of determining MMI dose recommendations based on FT4 measurements only. In detail, simulation studies show that although the patient-specific constant parameters of the established mathematical model may differ significantly from patient to patient, the input of TRAb is not necessarily required by the dosing algorithm. This is particularly important from a clinical point of view because according to current clinical practice, TRAb concentrations are only rarely measured during the course of MMI treatment. It is therefore only considered a minor limitation of our model that we did not differentiate between stimulating and blocking TRAb in our work.

Further aspects of the applicability of our mathematical model include the developed web application DigiThy (a first version is available at thyroid.tugraz.at) that can serve as a training tool for students and physicians in training. As it is easily accessible *via* our web application and easily comprehendible, it could provide a better understanding of hyperthyroidism and its MMI treatment. In brief, based on data including TSH, FT4, TRAb and previous MMI dose, a recommendation for the subsequent MMI dosing and the next appointment date can be chosen and entered by the trainee. Then the model calculates (predicts) the respective concentrations for the following appointment, at which this process can be reiterated. Our developed decision support system with its automatically generated MMI dosing recommendations could imply further-reaching improvements in GD management. The application of our mathematical model in clinical practice could standardize currently arbitrary treatment modalities in GD and could facilitate treatment for inexperienced physicians. CATT might eventually even render GD treatment more effective by helping to optimize MMI dosage and treatment time. Importantly, a future implementation of DigiThy into clinical routine may likewise improve clinical decision making and could be cost effective as it may reduce the requirement for consulting highly specialized endocrinologists.

Nevertheless, it is of course still premature to use DigiThy in routine patient care as this requires a clinical trial and a respective approval by health agencies. Therefore, to investigate whether treatment with our developed decision support system might be non-inferior or even more effective than current usual care, a randomized controlled trial (RCT) comparing usual care with CATT in real patients with GD is warranted. This RCT should be carried out in close collaboration between clinicians and control engineers to further refine and improve our model.

We are well aware that the underlying mathematical modeling of our work is challenging as it combines two very different disciplines, but open publishing of the entire framework of our approach is crucial to continue the development of our model and enable other groups to build upon our work. Future developments are needed as we are currently facing certain limitations. These limitations include the fact that validation to date was only carried out in virtual patients.

Furthermore, FT3 was not taken into account in view of the long-term course of treatment and modeling. An important point was the comparison between model performance and real patient data. A series of measurements was available for each patient, with usually several days or weeks between follow-up appointments. From medical and biological knowledge, it is known that the half-life of FT4 is several days, while the half-life of FT3 is usually about one day (33). In relation to the long intervals between follow-up appointments, the half-life of FT3 is far too short to make accurate statements about FT3 time behavior. While this fact already significantly complicates the long-term modeling of FT3, another difficulty comes in the form of the circadian rhythm of FT3. Such a circadian rhythm has been observed for FT3 but not for FT4. Thus, the time of day of the blood sampling may strongly influence FT3 measurements, but not FT4 (33). Hourly FT3 fluctuations could strongly influence the dosing until the next follow-up appointment (normally about 28 days) which is why it should not be incorporated in the CATT calculations. Furthermore, especially in primary care, it is sometimes common practice to only measure TSH and FT4 for economic reasons. Therefore, we think that only including FT4 in the proposed model enhances its applicability in clinical routine. However, we would like to stress that the clinical relevance of FT3 still needs to be considered by the treating physician when using CATT as a recommender system.

Also note that thyroid size was not explicitly considered, but effects of it arise implicitly from the parameters of the equations. Since the maximum synthesis excitation is given by ka1 and ka2 and these parameters are individual for each patient, the thyroid size can be implicitly considered *via* these terms. This was decided with model simplicity and applicability in mind, as thyroid sonography is not always available, especially when considering primary care.

Recently, a well-validated score (GREAT score) was established to assess the risk of disease recurrence in GD patients (34). While it proved to be a useful clinical tool for this purpose, it does not add any additional information for antithyroid drug dosage calculation and was therefore not considered for our proposed model. However, we would like to emphasize its benefit in clinical practice and individualized patient treatment.

Another limitation is that the differentiation of blocking and stimulating TRAb has not been available to date. Further, measurements such as TRAb are currently missing at many time points in our real-patient data owed to the nature of the study (registry study) they were derived from. Furthermore, currently, treatment recommendations are only possible for MMI, but not for alternative ATDs such as propylthiouracil. As thyroid hormones are significantly associated with heart rate (35), it is also tempting to speculate that integrating heart rate data obtained by common wearables such as smartwatches into our DigiThy application may also further improve our model, not only for guiding MMI dosing recommendations but also for detecting (re-) emerging hyperthyroidism. We also have to acknowledge that our work can only be considered a pilot study providing preliminary data on the characteristics and performance of our model and web application. We are well aware that the comparison of FT4 predictions by DigiThy to data of only 9 real patients can only be regarded as a rough proof of principle. These limitations should, however, be seen in the light of a unique software development with a great potential for a significant impact on daily routine care of millions of GD patients worldwide.

In conclusion, we developed a promising mathematical model and incorporated it into a web-based application software (DigiThy) serving as a training tool and as a CATT method for MMI dosing recommendations to improve GD patient care in the future. Further improvements of this approach and a clinical trial are required to evaluate the potential of this DigiThy application for routine care of patients with GD.

## Data Availability Statement

The original contributions presented in the study are included in the article/**Supplementary Material**. Further inquiries can be directed to the corresponding author.

## Ethics Statement

The studies involving human participants were reviewed and approved by the Ethics committee of the Medical University of Graz, Austria. The patients/participants provided their written informed consent to participate in this study.

## Author Contributions

VT-S, TB, CT, SP, and MR conceived and planned the study. TB and MR developed the mathematical model. VT-S, CT, and SP recruited the patients. VT-S, CT, and SP treated the virtual patients. VT-S, TB, and MR analyzed the data. VT-S, SP, and MR took the lead in writing the manuscript. All authors provided critical feedback and helped shape the research, analysis and manuscript.

## Conflict of Interest

The authors declare that the research was conducted in the absence of any commercial or financial relationships that could be construed as a potential conflict of interest.

## Publisher’s Note

All claims expressed in this article are solely those of the authors and do not necessarily represent those of their affiliated organizations, or those of the publisher, the editors and the reviewers. Any product that may be evaluated in this article, or claim that may be made by its manufacturer, is not guaranteed or endorsed by the publisher.
