# Supplementary material for: Mathematical Modeling of Free Thyroxine Concentrations During Methimazole Treatment for Graves’ Disease: Development and Validation of a Computer-Aided Thyroid Treatment Method
Source: Front Endocrinol (Lausanne). 2022 May 31;13:841888. doi: 10.3389/fendo.2022.841888 (PMC9205409; doi:10.3389/fendo.2022.841888)
Supplement: Supplementary file 1 [file Table_1.docx]

Supplementary Material

| patient  id | *J*u*,*1  usual care | *J*d*,*1  CATT | *J*u*,*2  usual care | *J*d*,*2  CATT | *J*u*,*3  usual care | *J*d*,*3  CATT |
| --- | --- | --- | --- | --- | --- | --- |
| 1 | 3.437 | 1.713 | 3.945 | 2.063 | 1.699 | 0.467 |
| 2 | 3.130 | 4.159 | 4.6 | 5.17 | 1.859 | 2.892 |
| 3 | 1.654 | 1.487 | 4.620 | 4.435 | 0.222 | 0.675 |
| 4 | 2.911 | 1.513 | 4.338 | 2.946 | 1.250 | 0.397 |
| 5 | 3.939 | 2.686 | 5.476 | 4.651 | 2.802 | 1.821 |
| 6 | 5.175 | 3.826 | 5.651 | 4.346 | 3.014 | 1.963 |
| 7 | 5.298 | 3.877 | 6.038 | 4.727 | 3.127 | 1.886 |
| 8 | 6.103 | 4.250 | 6.737 | 4.780 | 3.995 | 2.221 |
| 9 | 5.142 | 4.555 | 5.916 | 5.339 | 3.009 | 2.769 |
| 10 | 3.483 | 2.864 | 3.872 | 3.208 | 2.003 | 1.376 |
| 11 | 6.263 | 4.797 | 7.173 | 5.828 | 4.228 | 3.074 |
| 12 | 3.721 | 2.901 | 4.514 | 3.460 | 1.938 | 1.160 |
| 13 | 4.800 | 2.746 | 5.181 | 2.934 | 2.652 | 1.305 |
| 14 | 6.907 | 3.381 | 7.201 | 3.643 | 4.724 | 1.500 |
| 15 | 3.927 | 3.901 | 5.278 | 4.942 | 2.791 | 2.765 |
| 16 | 3.027 | 1.240 | 3.513 | 1.578 | 1.243 | 0.176 |
| 17 | 2.294 | 1.325 | 4.215 | 3.572 | 0.562 | 0.041 |
| 18 | 5.256 | 3.894 | 6.207 | 5.092 | 3.407 | 2.084 |
| 19 | 7.077 | 5.876 | 7.830 | 6.810 | 5.170 | 4.193 |
| 20 | 5.556 | 4.302 | 6.121 | 4.533 | 3.363 | 2.241 |
| 21 | 5.321 | 3.936 | 6.118 | 4.824 | 3.267 | 1.968 |
| 22 | 5.198 | 4.113 | 5.780 | 4.982 | 3.156 | 2.079 |
| 23 | 6.221 | 5.182 | 7.365 | 6.347 | 4.429 | 3.829 |
| 24 | 4.169 | 2.944 | 4.768 | 3.877 | 2.182 | 1.295 |
| 25 | 2.427 | 1.329 | 3.688 | 2.695 | 0.987 | 0.043 |
| 26 | 5.424 | 4.674 | 6.342 | 5.656 | 3.287 | 2.661 |
| 27 | 5.127 | 4.870 | 5.713 | 5.368 | 3.052 | 3.075 |
| 28 | 5.395 | 4.098 | 5.771 | 4.446 | 3.323 | 2.250 |
| 29 | 4.615 | 4.313 | 5.895 | 5.594 | 3.509 | 3.416 |
| 30 | 7.064 | 6.293 | 7.816 | 7.133 | 5.240 | 4.688 |
| 31 | 5.465 | 6.097 | 6.145 | 6.726 | 4.260 | 4.599 |
| 32 | 4.211 | 3.883 | 6.336 | 6.295 | 2.801 | 2.504 |
| 33 | 7.821 | 2.806 | 8.674 | 3.199 | 5.963 | 1.132 |
| 34 | 4.993 | 4.108 | 6.191 | 5.494 | 3.010 | 2.413 |
| 35 | 5.914 | 3.877 | 6.566 | 4.011 | 3.857 | 1.785 |
| 36 | 3.107 | 2.377 | 4.439 | 3.723 | 1.455 | 1.262 |
| 37 | 5.462 | 4.725 | 6.265 | 5.465 | 3.266 | 2.665 |
| 38 | 5.575 | 4.954 | 6.671 | 6.086 | 4.017 | 3.664 |
| 39 | 3.371 | 2.390 | 4.802 | 3.818 | 1.671 | 0.800 |
| 40 | 4.948 | 4.697 | 5.898 | 5.566 | 3.592 | 3.663 |
| 41 | 2.740 | 2.117 | 4.225 | 3.618 | 1.272 | 1.153 |

1. Supplementary Table 1: Achieved costs for 41 patients treated with usual care versus automated CATT procedure.
